# Knowledge on breastfeeding and improving cardiometabolic disease following a major complication of pregnancy: A qualitative analysis

**DOI:** 10.1177/17455057251366819

**Published:** 2025-08-30

**Authors:** Maleesa M. Pathirana, Emily Aldridge, Prabha H. Andraweera, Katie Lowe, Melanie Wittwer, Susan Sierp, Gustaaf Dekker, Margaret A. Arstall

**Affiliations:** 1 Adelaide Medical School, The University of Adelaide, SA, Australia; 2 Robinson Research Institute, The University of Adelaide, SA, Australia; 3 Department of Cardiology, Lyell McEwin Hospital, Elizabeth Vale, SA, Australia; 4 Division of Women’s Health, Lyell McEwin Hospital, Elizabeth Vale, SA, Australia

**Keywords:** breastfeeding, pregnancy complications

## Abstract

**Background:**

Maternal complications of pregnancy such as preeclampsia and gestational diabetes are independent risk factors for developing premature cardiovascular disease. Breastfeeding may improve immediate cardiometabolic health in these patients; however, women with pregnancy complications are less likely to initiate breastfeeding and more likely to cease breastfeeding early. It is still not known if women understand that breastfeeding can improve cardiovascular disease risk following a pregnancy complication, and if this knowledge would influence breastfeeding outcomes.

**Objectives:**

To assess women’s awareness of breastfeeding and cardiovascular disease risk reduction following a pregnancy complication.

**Design:**

Qualitative, descriptive study.

**Methods:**

Women with previous complications of pregnancy completed self-administered questionnaires and attended focus group style or one-to-one interviews at a tertiary hospital in Adelaide, South Australia. The following themes were discussed: experience with breastfeeding following a complication of pregnancy, knowledge on the benefit of breastfeeding for reducing heart disease, support for women to breastfeed for 6 months postpartum and integrated support during postpartum cardiovascular risk counselling. Interviews were transcribed, and deductive thematic analysis was undertaken with NVIVO V12.

**Results:**

Eight women attended interview sessions, with all women being aware that breastfeeding improves heart disease risk factors. However, only 75% of women knew that breastfeeding was particularly beneficial for women with a previous complication of pregnancy. Women reported attachment and guilt as major barriers to breastfeeding, and that breastfeeding support could be improved with individualised counselling prior to discharge, more frequent postpartum visits and explaining that breastfeeding can reduce cardiometabolic disease.

**Conclusion:**

These patient-reported barriers and areas of improvement are important to consider when tailoring lactation counselling support for women with previous complications of pregnancy.

## Plain language summary

**Women’s understanding of breastfeeding and improving heart health after a pregnancy complication**Women who experience a complication of pregnancy like preeclampsia or gestational diabetes have a greater risk of developing heart disease later in life. Breastfeeding for at least six months can reduce this risk, but women who have complications of pregnancy often also experience difficulties breastfeeding. We asked eight women who delivered their babies from 2021–2022, experienced a major complication of pregnancy and attended the postpartum lifestyle clinic for women with pregnancy complications at the Lyell McEwin Hospital what they knew about their risk of developing heart disease and how breastfeeding can help. We also asked what challenges they faced while breastfeeding, how they overcame these challenges and how women might be supported in future. Before the interviews, women were also given a questionnaire asking about their breastfeeding experience and understanding of breastfeeding and heart health. All interviewees agreed that ‘breastfeeding for at least 6 months can improve a mother’s blood pressure, weight and blood sugar’, however, only six agreed that this was also true for women who experienced a hypertensive disorder of pregnancy of gestational diabetes. Further, only six women had a health practitioner recommend breastfeeding for their health and not only to benefit their babies. Women reported a mix of positive and negative experiences about breastfeeding. The most common positive experience was women bonding with their baby. Women experienced the most common difficulty with getting an effective latch, especially when their babies were in the special care nursery. Women reported that the support they received that was beneficial and received by a variety of resources, including midwives, lactation consultants, family health nurses and online resources. Overall women reported that more support was needed greater support from health care professionals before leaving the hospital to improve breastfeeding confidence.

## Background

Over 30% of Australian women experience a pregnancy complication, with a higher rate in socioeconomically disadvantaged communities.^1^ Women with previous pregnancy complications, such as hypertensive disorders of pregnancy (HDP), gestational diabetes mellitus (GDM), placental complications (i.e. spontaneous preterm delivery and delivery of a small for gestational infant) have a two- to three-fold increased risk of developing premature cardiovascular disease (CVD) than those who have had an uncomplicated pregnancy.^2,3^ Therefore, preventative measures are required in the postpartum period to prevent the development of premature coronary artery disease in this high-risk cohort.

Breastfeeding has direct benefits for maternal cardiovascular health, with the length of breastfeeding associated with a moderate but significant reduction of CVD over time in the general population.^4^ Cohort studies have reported the effect of breastfeeding duration on improving postpartum cardiometabolic outcomes in women with complications of pregnancy.^5–7^ Therefore, supporting women with previous pregnancy complications to breastfeed may be beneficial for immediate cardiometabolic health and reducing risk of overall CVDs.

Women who have had pregnancy complications are more like to have difficulty with breastfeeding. The prevalence of delayed lactogenesis in women with previous GDM is 35%, with this prevalence being two- to three-fold higher amongst women who are also primiparous, treated with insulin in pregnancy and of advanced age.^8^ A history of HDP is linked with increased odds of non-exclusive breastfeeding at 4 months postpartum and low milk supply compared to those with normotensive pregnancies.^9^ Women with placental pregnancy complications are faced with physical barriers due to the increased likelihood of caesarean section and infant separation, which are both factors associated with inability to latch and breastfeeding cessation.^10^

Knowledge and beliefs around breastfeeding contribute to willingness to initiate and cease breastfeeding. Women who have had pregnancy complications are generally unaware of the link between their pregnancy complication and future CVD risk, with women reporting that knowing this would empower them to make better lifestyle choices.^11^ To our knowledge, beliefs around breastfeeding and CVD risk reduction following a major complication of pregnancy have not been explored, and it is unknown whether informing women about this association would influence breastfeeding decisions. Furthermore, we still do not fully understand the patient-reported barriers to breastfeeding in this high-risk cohort.

This study aims to determine the knowledge of women with previous pregnancy complications regarding breastfeeding and future CVD and determine the barriers to breastfeeding initiation and continuation in the same cohort.

## Methods

### Study design and setting

This is a qualitative descriptive study of women who attended a postpartum cardiovascular preventative clinic at the Lyell McEwin Hospital (LMH), South Australia. The LMH is a tertiary hospital servicing the Northern Adelaide Local Health Network, a population which has some of the highest rates of coronary artery disease, diabetes, smoking and obesity in metropolitan Australia.^12^ The LMH provides obstetric care, adult cardiac and intensive care services, and neonatal special care for infants ⩾32 weeks’ gestation. The LMH Women’s Health Department has ~4000 births annually. More than 50% of women who deliver at this hospital experience a major complication of pregnancy, with GDM being the most prevalent. This is higher than the national average of 25%–30%.^1^

Women were recruited from the LMH postpartum cardiovascular preventative clinic for women with major complications in pregnancy, described previously.^12^ In brief, women who have had a moderately–severely complicated pregnancy are referred to a nurse-practitioner-led, lifestyle intervention clinic for assessment of postpartum cardiometabolic health. Women who have any of the following complications are referred to this service:

- HDP [including gestational hypertension, preeclampsia, eclampsia and Hemolysis (red blood cell breakdown), Elevated Liver enzymes, and Low Platelet syndrome], requiring medical therapy or resulting in delivery at <37 weeks’ gestation.
- GDM requiring metformin or insulin therapy.
- Preterm delivery (<34 weeks’ gestation).
- Delivery of a small-for-gestational-age infant (<10th percentile).
- Placental abruption.

Ethics approval was received by Central Adelaide Local Health Network Human Research Ethics Committee (18875). Written informed consent was obtained prior to participation in the study. The reporting of the study conforms to the COREQ statement.^13^

### Data collection

Women who attended the postpartum cardiovascular clinic appointment at 6 months postpartum were invited via telephone call to participate in either a qualitative focus group with a brief questionnaire component or one-to-one interview session between June and August 2024, in the Clinical Trials Unit at the LMH. Potential participants spoke to a member of the postpartum clinic service team who told them that they were interested in understanding more about barriers to breastfeeding after a pregnancy complication. To start, women were given a questionnaire comprising two components:

- Questions about breastfeeding length and support received while breastfeeding.
- Current understanding of benefits of breastfeeding on general health and cardiovascular health.

After completing the questionnaire, the discussions were facilitated by a research team member (M.M.P.) with experience in qualitative study facilitation and a scribe (K.L.), who was responsible for audio recording, creating field notes during the interview and transcribing the interviews. The questionnaire and interview guide were developed by the study team, with the following key themes explored:

- Experience with breastfeeding after a pregnancy complication.
- Knowledge of the benefit of breastfeeding on future heart health following a pregnancy complication.
- Support for women with pregnancy complications to breastfeed for 6 months.
- Support to breastfeed at their 6-month postpartum cardiovascular appointment.

This interview guide was informed by the Health Belief Model (HBM)^14^ as a sensitising framework to explore the factors influencing breastfeeding intentions and barriers. The HBM constructions (i.e. perceived benefits of breastfeeding reducing CVD risk, perceived barriers to breastfeeding after a pregnancy complication and cues to action such as considering breastfeeding future children) were utilised to design open-ended questions to help understand women’s motivations and challenges around breastfeeding following a pregnancy complication.

### Qualitative and thematic analysis

All interviews were audio-recorded and transcribed verbatim. The interview transcripts and qualitative data from the questionnaires were exported to NVIVO V12 (Lumivero),^15^ a qualitative coding and analysis software. Deductive thematic analysis was conducted whereby preliminary themes were preconceived based on pre-existing material and theoretical framework.

Interview transcripts were read, with linkages across the transcripts coded back to specific themes. Data saturation was considered when theoretical saturation was reached and no new concepts or subthemes were identified from successive reviewing and coding. We reported coding density as the number of participants whose data were coded at each node. Theoretical saturation was reached for this study with eight women.

The preliminary themes were then analysed to create subthemes based on the coding for each theme. The meanings of the subthemes were synthesised into descriptions, with verbatim quotes from interview transcripts identified to illustrate responses relevant to each theme and description, where appropriate. Coding was validated through researcher triangulation; whereby coding analysis between two researchers was compared and discrepancies were resolved through refinement and discussion.

### Statistical analysis

Demographic data were analysed on IBM SPSS 27. Data were reported as either mean (range) or n (%). Socioeconomic status was defined by the Australia Bureau of Statistics Socio-Economic Indexes for Areas Index of Relative Socio-Economic Disadvantage (SEIFA IRSD).^16^

## Results

A total of eight women attended a focus group or one-to-one interview session (Table 1). All women experienced a major complication of pregnancy: 7 (87.5%) with HDP (5 (62.5%) cases of preeclampsia and 2 (25%) of gestational hypertension); 3 (37.5%) with GDM and 3 (37.5%) with preterm delivery. The average length of breastfeeding in the cohort was 9 months (range: 1.5–25 months; Table 2). All women were White (100%). The mean age of the cohort was 31.7 years (range: 27–38 years), and the mean SEIFA IRSD was 963 (range: 754–1023), which is lower than the state-wide and national averages.^12^ The majority of women had a bachelor’s degree (62.5%) and were employed part-time (75%). Half of the women had a combined annual household income of $70k–105k (50%). Six women reported having an extended hospital stay due to caesarean delivery (n = 6), preterm delivery (both spontaneous and iatrogenic due to preeclampsia; n = 3) and re-admission for preeclampsia (n = 1).

**Table 1.** Individual study participant characteristics.

Participant ID	Age (years)	Pregnancy complication(s)
P1	27	PE, sPTB
P2	31	PE, GDM, PTB
P3	31	PE
P4	36	GHTN
P5	38	PE
P6	31	PE, GDM
P7	29	GHTN, GDM
P8	31	GDM, PTB

GDM: gestational diabetes mellitus; GHTN: gestational hypertension; PE: preeclampsia; PTB: preterm birth; sPTB: spontaneous preterm birth.

**Table 2.** Demographics of study participants.

Age	31.7 (27–38)
SEIFA-IRSD	963 (754–1023)
Income level^a^	
$70,000–105,000	4 (50%)
$105,000–205,000	2 (25%)
>$200k	1 (12.5%)
Education level	
Bachelor	5 (62.5%)
Certificate	2 (25%)
Diploma	1 (12.5%)
Employment	
Part-time	6 (75%)
Maternity leave	2 (25%)
Complications experienced^b^	
GDM	3 (37.5%)
HDP	7 (87.5%)
PET	5 (62.5%)
GH	2 (25%)
Preterm delivery	3 (37.5%)
Breastfeeding length (months)	9 (1.5–25)

Data reported as mean (range) or n (%).

GDM: gestational diabetes mellitus; GH: gestational hypertension; HDP: hypertensive disorders of pregnancy; PET: preeclampsia; SIEFA-IRSD: Socio-Economic Indexes for Areas Index of Relative Socio-Economic Disadvantage.

a n = 7.

b Pregnancy Complications (PC) are not mutually exclusive.

### Experience with breastfeeding

Overall, no woman had a straightforward or simple breastfeeding experience (Supplementary Figure 1). There was a mix of both positive and negative experiences. For those who had positive experiences, the most common was enjoying bonding with their baby:

The one thing I look back and I was like “I loved that” was when I was able to start breastfeeding. I’ve literally got videos and photos on my phone of my first breastfeed and my last because that was so special to me. . . I really treasure those videos. (P1)

Women felt that once they overcame challenges, they could then fully enjoy the experience:

It [breastfeeding] was very tiring, but once he actually got his latch, everything was a lot better. (P2)

Women reported various negative experiences of their breastfeeding journey, with two subthemes being the most prevalent:

#### Attachment issues and expressing

All women reported physical attachment issues and expressing as negative experiences.

Women commented on difficulties with attachment due to physical separation from their babies:

It was very rocky at the beginning, with the breastfeeding. I had to work really hard to kind of keep going with it. Because [my baby] was in special care. . . it took about a week, I think to get my first initial latch with her. (P1)

Six women (75%) commented that expressing breastmilk caused significant fatigue, both physically and mentally:

Yeah, you’re expressing overnight, and you don’t have the baby there with you. That was probably the hardest part. (P3)

I think I was pumping every two hours, even overnight. . . I had a c-section, [and was] trying to look after a baby and pumping. . . I felt like I had no time to myself or to decompress. (P4)

Where’s my time, like, where do I get a break? Where do I get to have a shower? Yeah, it was just, it was a lot. (P5)

#### Pressure and guilt around breastfeeding

Six women (75%) reported that they felt extreme pressure to breastfeed. Some women felt they were failing their baby when they faced issues with low supply:

. . . [the midwives would say] “you need to breastfeed. You need to breastfeed”. . . [it felt more like they were saying] “you need to try harder. Because your baby’s not getting what they need. He needs your breast milk . . .” (P7)

I was getting stressed because I wasn’t producing enough [breastmilk]. She was getting hungry, it was making me feel really upset. (P5)

Some women believed that the culture to breastfeed as the preferred feeding option within the antenatal wards contributed to them feeling pressured:

I’ve got low supply. I tried to go in with like realistic expectations for myself . . . when [you work as] a midwife you can fixate on things a bit more. (P3)

I was so upset [that] I couldn’t breastfeed to start off with . . . you hear about it all the time when you’re here, like breastfeeding’s the best way to do it and even though they weren’t being pushy as such, you put that pressure on yourself. (P4)

There’s a lot of “breast is best,” [posters and signs] I recall in the, in the rooms . . . there were a lot of signs about focusing on it [breastfeeding] in the room and you can’t help but like see it all the time. . . it’s just very in your face [confronting]. (P5)

### Support received to overcome the challenges of breastfeeding

#### Antenatal nurses and midwives

Nearly all women (87.5%) reported that they overcame challenges with breastfeeding with the help of the antenatal team (Supplementary Figure 2). Many women commented on the expertise of the midwives:

. . . they [the midwives] were very supportive . . . I couldn’t get him on [the breast] properly despite my best efforts and one of the [midwives] . . . came in and said “oh, why don’t we try a nipple shield?” . . . that was really useful. . . it bridged a bit of a gap for me . . . in between trying to get him on at the nursery and when we went home. . . (P3)

Midwives were able to provide encouragement to women:

They’re [midwives] just so relaxing and calming . . . they just know how to say the right thing to make you feel encouraged and you’re never judged, and they were always yeah very helpful with ideas. (P2)

. . . everything that they [the midwives] tried to do for me [to help me breastfeed], I felt, I was grateful for because they were trying to get me to that same spot I wanted to be in. (P6)

One woman reflected on the emotional support she received from the midwives amidst her post-delivery recovery:

I did probably have maybe two or three midwives that were absolutely like phenomenal. Like they sat there on the bed with me while I cried. (P7)

Women also commented on how beneficial it was to have a midwife on-call after delivery to help with breastfeeding problems:

[Having the midwives] sort of being on my beck and call [available to help] when baby was hungry, that was the time to show me what to do. . . In those early hours of the morning and then, you know, she’d check on me during my stays. (P6)

I had one midwife on, like the night she was born. . . they came as soon as I was awake and brought her [my baby] to me and tried to latch her and tried to help me express her first kind of bit of milk from me. (P1)

#### Family health nurses

Half of the women reported that they sought support from family health nurses for breastfeeding in the postpartum period. Nurses were helpful with assessing women at home while they were feeding to identify an immediate solution to any breastfeeding problem:

I did have a bit of help with the CAFHS [Child and Family Health Service] nurses, as well when they came by, especially when he [my baby] was learning to attach, so they were helping with that. (P2)

I do remember . . . one [nurse] coming and she helped me like sit on my bed, because that’s where I was feeding. She was showing me like how to prop pillows up and support myself and support my baby. So yeah, definitely, that was helpful. (P5)

#### Lactation consultants

Lactation consultants (LCs), seen on the hospital ward and in the postpartum period, were beneficial in overcoming breastfeeding challenges. Although only two women reported on the questionnaire that they sought an LC for breastfeeding support (Table 2), at least 50% reported receiving support from an LC, including on the ward.

One woman commented on how the LC took time to assess their issues:

I think I was there for maybe two hours with her [the LC], and got a lot of information from her . . . Lactation consultants I think are definitely very, very helpful. (P4)

Women reported that LCs were very useful at finding the best feeding positions:

I think positions [to hold my baby while feeding] probably was a big thing for me . . . they showed me some different holds that can kind of better support her kind of suck-swallow and everything that helped me breastfeed. (P1)

I had the lactation consultants come show me different ways of holding [my baby while breastfeeding] and all that sort of stuff. (P6)

#### Online resources

Women also sought their own resources online, which they felt allowed them to overcome certain challenges. Online videos and social media pages provided women with a visual medium that helped them overcome problems with expressing and attachment:

[Accessing online resources] . . . was really good because [the video] was really engaging, and it was all videos and it was like watching and learning and how she would um describe things. And uh I guess demonstrations on how to do it. So it was easy to follow. (2)

She [the video presenter] did reviews on all different breast pumps and things, so I learnt a lot about different breast pumps and options and how to check that your flanges fit correctly and all that through her, so, yeah, it was really interesting. (P4)

Women also felt that online resources allowed them to then go on to find support in person:

Obviously, when things [breastfeeding difficulties] were happening at home, I would look it up online and figure it out and then have a chat with them [health professionals] about what I’ve read online, and see where they were coming from. (P8)

I kind of just Googled [my breastfeeding problems], to be honest, what was going on and then that, kind of told me to perhaps seek an IBCLC [LC] so that’s when I went ahead and did that. (P1)

One woman commented on how their online community gave them a sense of support:

No one else in any of my mum’s groups . . . had exclusively pumped [breastmilk to bottle feed their baby] so I didn’t have anyone directly that I could talk to. So having that online community was the support that I needed, and [helped with] knowing I wasn’t alone. So I think that’s what helps. (P5)

### Knowledge around breastfeeding and future heart health

All women (100%) knew that breastfeeding for at least 6 months could improve cardiovascular risk factors like blood pressure and cholesterol. However, only six women were aware that breastfeeding was particularly beneficial after having an HDP or GDM (75%). Additionally, six women reported that a health practitioner told them of the benefits of breastfeeding for improving overall health (Table 3).

**Table 3.** Breastfeeding questionnaire.

Support for breastfeeding	
Midwife/nurse	7 (87.5%)
Lactation consultant	2 (25%)
CAFHS	4 (50%)
Medication	1 (12.5%)
GP	1 (%)
Family/friend	2 (25%)
Online resources	2 (25%)
True or false	
Breastfeeding for at least 6 months can improve a mother’s blood pressure, weight, cholesterol and blood sugar	True: 8 (100%) False: 0 (0%)
Breastfeeding for 6 months after having preeclampsia or gestational hypertension can reduce a mother’s heart disease risk	True 6 (75%) False: 2 (25%)
Breastfeeding for 6 months after having gestational diabetes can reduce a mother’s heart disease risk	True (75%) False: 2 (25%)
Did any health practitioner recommend that you breastfeed to improve your overall health or any aspect of your health (not only to feed your baby)?	Yes (75%)
If yes who?	
GP	3 (37.5%)
Lactation consultant	2 (25%)
Midwife/nurse	2 (25%)

Data reported as n (%).

CAFHS: Child and Family Health Service; GP: general practitioner.

Only three women (37.5%) reported that they had some idea about breastfeeding improving heart disease risk after a pregnancy complication, with two women reporting knowing this from their line of work:

I just know that [breastfeeding] that does help lower your risks and whatnot. And I think if you keep breastfeeding for longer than six months, it helps a lot further. It’s obviously – breastfeeding is really important for mum as well as baby. (P2)

Women did know about the benefits for their baby:

Oh, I can’t remember any of that [about breastfeeding being beneficial for the mother]. All I know is that, from what I’d heard, breastfeeding is the best thing for the baby. (P8)

Women knew about other health-related benefits:

I knew that there were health benefits [of breastfeeding]. I think the one I knew mainly was something to do with cancer? Decreases risk of breast cancer, I think. But not really a lot else about it to be honest. (P4)

I knew [breastfeeding was] good for like general health and everything, but not specifically for heart-related things. (P1)

Similarly, for women who had GDM, they did not know much about how breastfeeding can reduce the risk (or defer onset) of type 2 diabetes:

I don’t know if they specifically said that it [breastfeeding] can lower my risk of type 2 diabetes or anything like that. (P2)

Women were additionally asked “now knowing about the benefits of breastfeeding on heart health following a complicated pregnancy, does this influence your future decision to breastfeed or would you try to breastfeed if you were to have another child?” All women agreed that they would breastfeed again for a future baby, but their maternal benefits were not the primary reason:

The health benefits [for me] probably weren’t the primary reason why I did it [breastfed my baby]. It was more like a mental achievement and, and knowing that I was able to do it and provide the best I could for [my baby], that made it my reason to keep doing it and that’s why I want to do it again. (P4)

One woman commented that they would try again but know their own personal limits to breastfeeding:

Yeah, I want to give it [breastfeeding] a go. I’m still going to try and give it a go but I’m just gonna [going to] go in there with a bit more of a mindset of what my body can do and when I know to stop. (P6)

### Support required for women to breastfeed following a pregnancy complication

Overwhelmingly, all women identified that women required more support to breastfeed successfully. One woman commented on how this is needed for all mothers, regardless of experience:

You could be a first-time mum not getting that extra assistance that they need, and then you could be, you could have two or three kids, and then people assume you know exactly what you’re doing and don’t provide that support that you might need. (P3)

Women felt that greater frequency in support was needed in postpartum:

I had lots of help [with breastfeeding] in the hospital. . . but it was more looking for it myself after I left the hospital, like [attending] follow-up appointments and me voicing that I needed help or, booking my own GP appointments to ask what else I can do? (P6)

I don’t know if [support] can be at home because it’s hard for women to leave home to come in [to an appointment], especially a couple weeks post [delivery] . . . [arranging for] someone to come out and educate them or maybe just a phone [call]. (P2)

Women felt that immediate, individualised support post-delivery was needed to assess any issues before discharge:

. . . [Professionals need to see women] 48 to 72 hours [after delivery], when they’re in the hospital . . . sometimes it’s just a little thing that you need to do, to tweak, to change what’s happening. (P3)

You need that one-on-one with someone [on the ward], where you can sit down for half an hour and go through it [breastfeeding] together. (P5)

Two women commented on the lack of consistency for support, identifying that this was dependant on a woman’s length of hospital stay after delivery. They commented further on how they were fortunate to receive the support they did because of the length of time they were admitted:

I know having those five days in hospital [was beneficial] for me. They were invaluable because I could just focus on trying to get that routine started with my little one and to start that breastfeeding. (P5)

I spent at least one to two weeks in the NICU with all of mine [my children]. So I didn’t have . . . by the time I was discharged, or my kids were discharged . . . They were feeding fine. And I don’t have those issues. (P8)

Five women (62.5%) reported that they wanted the opportunity to see an LC on the ward before they were discharged:

I’d love to have a lactation consultant come . . . to see the mums like immediately after birth . . . to see someone that could check for the tongue ties straight up, make sure that was all good and like kind of help you with positions and everything like that from the get-go would be really beneficial. (P1)

I think having like an extra lactation consultant that could pop in and just say “hello, any issues?” Just to provide that opportunity um for women to say, “I’m having these issues” [would be beneficial]. (P3)

Two women commented that they did not know that an LC was available on the ward to support women with breastfeeding:

I don’t know if there actually are any [LCs] here that come in the hospital, that come to see you while you’re here, because I definitely didn’t see one. (P4)

Women reported that the hospital midwives were very helpful but acknowledged they were busy and that an LC visit could provide more support:

Yeah, because the nurses and the midwives are so busy doing everything else, they can’t be expected to be lactation consultants as well. I know some of them probably do have specialty in that kind of area as well . . . (P4)

. . . from what I’ve seen as a midwife, there is a massive lack of support for women on the ward, like, directly after they’ve had their baby . . . I think that’s due to shortages of staff . . . and the workload being too much for the midwives. I feel like currently the system sets [midwives] up to fail a little bit, because we just don’t have the time to give everyone what they need. (P3)

Conversely, women who worked within healthcare acknowledged that the LCs on the ward may also not always be available:

[The ward] don’t really have that [capacity to help with breastfeeding], so you can ring the lactation consultants and they might come if they’re free but they’re often very busy because their clinic’s always full. (P2)

. . . and I know midwives that work in [lactation consultant clinics] would love more hours but there’s just such limited resources . . . (P3)

Women were asked whether those with complications of pregnancy should have specific support, with 5 (62.5%) women reporting that this would be beneficial:

If there is a benefit, like, they can show a real benefit in the increase of breastfeeding to minimising complications with heart disease and stuff then yes, I think there should be additional support . . . As long as it’s not pressured additional support. You know, the option needs to be there, I think. (P4)

. . . I feel like when you’ve got those complications, that’s already sort of setting you out for things like delayed lactation and your mode of birth, having a caesarean and being limited with actually feeding your baby. Yeah, 100 percent . . . [that would be beneficial]. (P3)

In relation to preeclampsia, three women discussed that they were unaware of the severity of their condition:

I didn’t realise. . . how bad everything [my condition] was. . . there needs to be some sort of debrief. (P4)

I didn’t really know a lot about [preeclampsia] until it happened [to me]. But absolutely, next time around . . . I would love to have whatever support is available for sure. (P1)

One woman commented that due to the severity of their preeclampsia, they felt additional breastfeeding support may have been detrimental:

I don’t think I took preeclampsia as serious[ly] as I should have, I didn’t realise how serious [my condition was] . . . mentally, it could really play on people’s minds when it comes to feeding as well like that, maybe they’re feeling like their body’s failing them or, you know, they’ve done something wrong and that could have that flow-on effect. (P5)

Women commented that the timing of receiving information is also important:

I don’t think I ever really had someone sit down and talk to me about the benefits [of breastfeeding] specifically until I was in the [postpartum heart health] clinic. (P2)

If [information] is given maybe more preclinic, before actually giving birth [that might be more helpful] . . . so that if you’re on maternity leave for however long, or you’re pre-maternity leave, then you’ve got more time to read through stuff, you’ve got more time because you’re starting to get in that stage where you’re looking at what you can do to benefit everything, looking at what you can do best for baby, best for you. (P8)

### Implementing support in postpartum cardiovascular counselling

Six women (75%) agreed that women should be provided with breastfeeding support in the 6-month postpartum clinic for women with complicated pregnancies, but with the caveat that this should only be if a woman wants to continue their breastfeeding journey at this timepoint:

I think reinforcing [continuing to breastfeed], isn’t a bad thing. Explaining, why? Especially if they are still breastfeeding. If they have stopped for some reason, like they can’t do it anymore or some reason like that, it’d [encouraging breastfeeding would] probably be more harmful than good, but if they are still breastfeeding then it’s just a reinforcement that what you’re doing is the best thing for you, for baby, all of that. (P8)

. . . I think if they’re happy breastfeeding then definitely [clinic staff should be] supporting them. If they’re on the fence, for me, I would still support them, but let them know that either decision is fine. Like obviously there’s health benefits for it [breastfeeding] for both you and baby, but if you are ready to stop, then you should be proud of the achievement that you’ve made so far. (P7)

All women (100%) agreed that if a woman was willing to continue breastfeeding after their baseline appointment, then discussing how breastfeeding can improve their immediate cardiometabolic health would be very useful:

Yeah, I think so because it [talking about the benefits to my cardiometabolic health can been seen in my blood test results] makes it, like its real life. Like this is what’s happening. This is why your body . . . It’s proving that, you know, breastfeeding’s helping your body. . . for me that would help. (P2)

[Yes, it would be] A bit of an incentive. (P7)

Women collectively agreed that this counselling required the clinician to discuss breastfeeding at this time point in an encouraging and open manner:

I think it depends on the individual circumstances . . . So if I feel like I was in that person’s shoes and struggling or not sure to keep going [with breastfeeding], I would love a bit of encouragement. (P1)

## Discussion

The aim of this qualitative study was to explore the breastfeeding and future CVD risk knowledge of women who had experienced a complicated pregnancy. We found that all women who participated understood that breastfeeding was associated with a reduction in CVD risk factors; however, not all understood that breastfeeding could reduce heart disease risk following a pregnancy complication. Our secondary aim was to identify barriers to breastfeeding in this cohort. We found that the most common barriers were related to physical attachment and expressing, and external pressure and guilt.

While 100% of women knew about the benefits of breastfeeding for reducing heart disease risk factors, 25% did not realise that these benefits are amplified for women who have had a previous pregnancy complication. In a recent U.S. study of 451 pregnant women, only 25% knew that breastfeeding reduced the risk of hypertension and heart disease.^17^ Furthermore, women with a history of hypertension were less likely to strongly want to breastfeed compared to those who did not have a history of hypertension.^17^ Awareness of heart disease risk following a pregnancy complication is already low amongst women, our cohort included.^18^ Health professional knowledge of pregnancy complications and future cardiometabolic disease has been reported to be suboptimal, with obstetricians and antenatal nurses scoring moderate–low for knowledge on HDP and cardiometabolic disease.^19^ Furthermore, our study also identified that women who had GDM were not aware that breastfeeding reduced type 2 diabetes risk. A qualitative study on breastfeeding practices within Japanese hospitals for women with GDM identified that clinicians did not understand how to counsel women about reducing type 2 diabetes risk by breastfeeding; reporting inconsistency across staff regarding competency and knowledge in this area.^20^ Therefore, clinicians require greater education about breastfeeding for improving CVD risk following pregnancy complications in order to delivery consistent, high-quality care. Educating women on breastfeeding and improved cardiometabolic health during lactation counselling may be an influencing factor for a women’s intention to breastfeed and breastfeeding longevity, in conjunction with other personal factors, while concurrently improving awareness of future heart disease risk.

Our cohort of women with previous pregnancy complications reported two primary barriers to breastfeeding. First, attachment issues and expressing were reported as a barrier to breastfeeding by nearly all women. These patient-reported barriers are commonly reported in the literature from general populations of women.^21–23^ Six of the women in the study had an emergency caesarean delivery, which is already known to be associated with reduced ability to latch and low breastmilk supply. This is thought to be due in part to an increased maternal and foetal stress response and delayed skin-skin contact promoting delayed lactogenesis.^24^ Issues related to attachment are further complicated for women with placental-mediated complications, particularly from infant separation in the case of those delivering preterm or small-for-gestational-age infants, or maternal illness from severe HDP (e.g. HELLP syndrome). A randomised control trial by Mattar et al. provided individual antenatal lactation counselling focused on expressing and latching, finding that women had a two-fold higher odds of breastfeeding at 6 months postpartum.^25^ It is still unclear whether antenatal breastfeeding interventions are effective at improving breastfeeding longevity and maternal well-being^26,27^; however, it is known that positive encouragement and interventions improve breastfeeding self-efficacy.^28^ Therefore, providing antenatal counselling on attachment and expressing for women with pregnancy complications and poor labour indications, where practical, may improve breastfeeding initiation and self-efficacy.

Women also identified that they felt pressure and guilt as a barrier to breastfeeding successfully. Our women linked this strongly with poor mental health, which was a contributing factor in stopping their breastfeeding journey. A systematic review in 2021 identified that women with symptoms of postpartum anxiety had reduced self-efficacy of breastfeeding.^29^ Furthermore, our cohort reported that the pro-breastfeeding discourse at the hospital inadvertently placed pressure and subsequent guilt on them when they were unable to breastfeed. It has been reported that this guilt may be amplified by infant feeding initiatives, whereby women are not prepared for realistic postpartum challenges of breastfeeding.^21,30^ Therefore, antenatal education and breastfeeding support should continue to discuss breastmilk positively but also be wary of the negative connotation regarding “breast is best.” For women with complications of pregnancy who are known to have greater difficulty in initiating breastfeeding, approaching pro-breastfeeding discussions with appropriate support and education to begin their breastfeeding journey may allow women to feel fully prepared for potential challenges.

Our cohort reported a range of supports and mechanisms they felt could benefit women to be able to breastfeed successfully. Almost all (87%) of our cohort reported that post-delivery support from the antenatal team was extremely beneficial to overcome challenges. Meshram et al. reported that women who received both postnatal counselling and physical support had three-fold higher odds of initiating early breastfeeding, even with adjustment for socioeconomic factors and education status.^31^ Furthermore, LCs were highly praised and encouraged to be part of routine care in the postnatal period. This is in line with previous studies reporting the efficacy of lactation counselling on prolonged breastfeeding.^32,33^ Our women reported that immediate, individualised postnatal support was needed to identify issues before discharge from hospital. This period of time is crucial for women to establish breastfeeding and rectify any problems.

Women also felt that specialised breastfeeding support for women with complications of pregnancy was important. Women who have pregnancy complications are likely to be overweight/obese, have prolonged labour, require caesarean section and have social barriers (i.e. infant separation) to feeding compared to women with uncomplicated pregnancies, contributing to delayed lactogenesis.^34–36^ These factors may be important to consider in the postnatal period to improve initiation rates in this cohort. A systematic review identified that breastfeeding interventions supported women with previous GDM to exclusively breastfeed in the early postpartum (first 4 weeks), but these interventions supported non-exclusive breastfeeding only beyond 6 months.^37^ Therefore, breastfeeding interventions for this cohort need to be evaluated to determine how best to promote longevity of exclusive breastfeeding.

When asked if breastfeeding support should be provided at 6 months postpartum in our lifestyle clinic, all women felt that this must be individualised based on a mother’s current state in their breastfeeding journey. However, they did agree that for women who were still breastfeeding, discussing how breastfeeding can reduce cardiovascular risk was valuable. While breastfeeding is considered an important caveat in postpartum cardiovascular counselling,^38,39^ there is no consensus on how best to provide this advice, particularly as breastfeeding support is primarily driven by midwifery and antenatal nurses. Furthermore, it is important to acknowledge that while our participants felt that the maternal benefits of breastfeeding were an important factor in their decision to breastfeed, the emotional and personal benefits outweighed this. Therefore, it may be necessary to integrate antenatal education services, postnatal lactation support and CVD preventative counselling in a holistic and patient-centred manner to improve breastfeeding outcomes and reduce future CVD risk in this cohort.

Our study has a number of strengths. To our knowledge, this is the first Australian study with a cohort of women experiencing major complications of pregnancy exploring knowledge on breastfeeding and heart disease. The use of a qualitative design with a brief questionnaire component allowed us to receive richer information from participants as we were able to ask a greater variety of questions by using qualitative and quantitative methods. We recruited women from an outpatient cohort where the rate of pregnancy complications and heart disease is higher than the national average.^40^ Women who participated in the focus groups attended the same hospital service for both antenatal care and their postpartum follow-up, allowing their experiences to be specific to our service, which will improve continuous patient care.

### Limitations

As women in this study attended a postpartum specialist service, it is likely their health literacy and understanding of their future risk of heart disease are higher than the general population, thereby leading to selection bias. Furthermore, selection bias is evident, as participants were highly educated, were all White and had a mean breastfeeding length of 9 months. Although the cohort was drawn from a region of socioeconomic disadvantage (where breastfeeding rates are lower than in affluent socioeconomic regions), many participants reported access to resources and support, including postpartum CVD screening. Notably, 50% of our cohort could access paid lactation consultancy services. While our postpartum clinic does not directly provide lactation support, these participants reflect a cohort who are better equipped to seek and navigate breastfeeding challenges and may not entirely represent the broader population of disadvantaged women with pregnancy complications who may not have access to these resources.

Additionally, we only included English-speaking participants due to resource restraints regarding conducting focus groups with interpreters. Our findings may not be generalisable to those of non-White populations where breastfeeding rates are significantly lower.^41,42^ An Australian study of culturally and linguistically diverse patient experiences of exclusive breastfeeding identified that reasons for breastfeeding cessation included lack of prenatal intention to breastfeed, reduced partner support and depressive symptoms. These themes were not captured significantly throughout our study, which represents the contrasting experiences of both cohorts.

We must also acknowledge that while we have reached data saturation with eight participants, this is likely because our cohort is very homogenous in terms of their demographics and experiences. If we were to have had a larger sample with more diversity across our participant pool, we may have uncovered additional perspectives and themes that could not be uncovered from our current cohort.

We acknowledge that this lack of diversity excludes key perspectives and limits our findings to a very specific cohort of patients. Future studies should seek to intentionally sample more diverse populations, particularly those of different racial and ethnic minority groups to capture the full spectrum of breastfeeding experiences following pregnancy complications.

The questionnaire administered to study participants was used to guide and complement the data collected from the qualitative study, the questionnaire was not pilot-tested and may not be suitable to use across different populations.

## Conclusion

Women with previous complications of pregnancy were not generally aware that breastfeeding could improve cardiovascular and diabetes risk following such complications. Furthermore, this cohort identified that attachment/expressing and initiative-based pressure were major barriers to breastfeeding. Considering physical and psychosocial barriers specific to this cohort is necessary to tailor lactation counselling to improve breastfeeding outcomes and future cardiometabolic health. Optimised lactation counselling should be considered during antenatal care and tied in with postpartum CVD prevention for continuity of care in this high-risk cohort. Greater understanding of breastfeeding experiences among diverse patients, particularly ethnically and culturally diverse communities, is needed to develop targeted strategies to improve breastfeeding outcomes in these populations.

## Supplemental Material

sj-docx-1-whe-10.1177_17455057251366819 – Supplemental material for Knowledge on breastfeeding and improving cardiometabolic disease following a major complication of pregnancy: A qualitative analysis Supplemental material, sj-docx-1-whe-10.1177_17455057251366819 for Knowledge on breastfeeding and improving cardiometabolic disease following a major complication of pregnancy: A qualitative analysis by Maleesa M. Pathirana, Emily Aldridge, Prabha H. Andraweera, Katie Lowe, Melanie Wittwer, Susan Sierp, Gustaaf Dekker and Margaret A. Arstall in Women's Health

sj-docx-2-whe-10.1177_17455057251366819 – Supplemental material for Knowledge on breastfeeding and improving cardiometabolic disease following a major complication of pregnancy: A qualitative analysis Supplemental material, sj-docx-2-whe-10.1177_17455057251366819 for Knowledge on breastfeeding and improving cardiometabolic disease following a major complication of pregnancy: A qualitative analysis by Maleesa M. Pathirana, Emily Aldridge, Prabha H. Andraweera, Katie Lowe, Melanie Wittwer, Susan Sierp, Gustaaf Dekker and Margaret A. Arstall in Women's Health

sj-pdf-3-whe-10.1177_17455057251366819 – Supplemental material for Knowledge on breastfeeding and improving cardiometabolic disease following a major complication of pregnancy: A qualitative analysis Supplemental material, sj-pdf-3-whe-10.1177_17455057251366819 for Knowledge on breastfeeding and improving cardiometabolic disease following a major complication of pregnancy: A qualitative analysis by Maleesa M. Pathirana, Emily Aldridge, Prabha H. Andraweera, Katie Lowe, Melanie Wittwer, Susan Sierp, Gustaaf Dekker and Margaret A. Arstall in Women's Health
