# Supplementary material for: Knowledge on breastfeeding and improving cardiometabolic disease following a major complication of pregnancy: A qualitative analysis
Source: Womens Health (Lond). 2025 Aug 30;21:17455057251366819. doi: 10.1177/17455057251366819 (PMC12399822; doi:10.1177/17455057251366819)
Supplement: sj-docx-1-whe-10.1177_17455057251366819 – Supplemental material for Knowledge on breastfeeding and improving cardiometabolic disease following a major complication of pregnancy: A qualitative analysis [file sj-docx-1-whe-10.1177_17455057251366819.docx]

Initials: _______ Date: ______________

Breastfeeding questionnaire

**Part A:**

1. Did you breastfeed? YES NO
2. If yes, how long did you breastfeed for (Months/weeks) __________________
3. If you breastfed, did you receive breastfeeding support from any of the following services?

CAFHS  LACTATION CONSULTANT

MEDICATION  GROUP CLASSES

GP  FAMILY/FRIEND

MIDWIFE/NURSE  NONE

Other (please specify below)

If Other: _____________________________________________________

_____________________________________________________________

_____________________________________________________________

***Please turn over.***

**Part B: Please circle TRUE/FALSE/NOT SURE for each statement:**

1. Breastfeeding for at least 6 months can improve a mother’s blood pressure, weight, cholesterol, and blood sugar.

True False Not Sure

1. Breastfeeding for 6 months after having preeclampsia or gestational hypertension can reduce a mother’s heart disease risk.

True False Not Sure

1. Breastfeeding for 6 months after having gestational diabetes can reduce a mother’s heart disease risk.

True False Not Sure

Did any health practitioner recommend that you breastfeed to improve your overall health or any aspect of your health (not only to feed your baby)?

Yes  No

If yes, who?

🞎 OBSTETRICIAN 🞎 MIDWIFE/NURSE

🞎 GP 🞎 CARDIOLOGIST

🞎 LACTATION CONSULTANT

🞎 OTHER _______________________________

***This is the end of the questionnaire.***
