# Supplementary material for: Knowledge on breastfeeding and improving cardiometabolic disease following a major complication of pregnancy: A qualitative analysis
Source: Womens Health (Lond). 2025 Aug 30;21:17455057251366819. doi: 10.1177/17455057251366819 (PMC12399822; doi:10.1177/17455057251366819)
Supplement: sj-docx-2-whe-10.1177_17455057251366819 – Supplemental material for Knowledge on breastfeeding and improving cardiometabolic disease following a major complication of pregnancy: A qualitative analysis [file sj-docx-2-whe-10.1177_17455057251366819.docx]

Theme 1:
Experience with breastfeeding after a pregnancy complication (n=8)

Positive (n=7)

Negative experiences (n=8)

Led to improved bonding and sense of confidence (n=4)

Attachment issues (n=8)
N=6 participants with extended stay

Pressure and guilt around breastfeeding (n=6)

Participants overcoming these challenges (n=4)

Supplementary Figure 1: *Reconstructed figure depicting concepts emerging from Theme 1: Experience with breastfeeding after a pregnancy complication.*

Caption: Code hierarchy was developed through thematic analysis with NVIVO. Numbers in parentheses represent coding density, defined as the number of participants whose data were coded to at each node. Coding was conducted across five focus groups and individual semi-structured interviews.

(a) (b)

(c) (d)

Supplementary Figure 2: *Reconstructed figure demonstrating concepts emerging from Theme 2: Support received to overcome the challenges of breastfeeding; stratified by nodes (a) midwives (b) lactation consultants (c) family health nurses (d) online resources.*

Caption: Code hierarchy was developed through thematic analysis with NVIVO. Numbers in parentheses indicate coding density, defined as the number of participants whose data were coded to at each node. Coding was conducted across five focus groups and individual semi-structured interviews.
